# Hungry no More? The Joint Impact of Minimum Wages and the Earned Income Tax Credit on Food Insecurity

**DOI:** 10.1002/hec.70001

**Published:** 2025-06-10

**Authors:** Otto Lenhart, Kalyan Chakraborty

**Affiliations:** ^1^ Department of Economics University of Strathclyde Duncan Wing Strathclyde Business School Scotland UK; ^2^ Department of Business Lamar University Beaumont Texas USA

**Keywords:** earned income tax credit, food insecurity, minimum wage, poverty

## Abstract

In this study, we provide evidence on the combined effect of state minimum wages and state Earned Income Tax Credit (EITC) benefits on food insecurity. Using data from the Current Population Survey Food Security Supplement (CPS‐FSS) between 2001 and 2019 and a sample of individuals with at most a High School degree, we estimate difference‐in‐differences models to examine whether the policies have a joint impact on food insecurity. Our study adds to a small number of papers evaluating potential interactions between state minimum wages and EITC laws. Our analysis reveals the presence of joint effects of the two programs in terms of reducing food insecurity. We find that a $1 increase in minimum wages reducing the likelihood of households experiencing very low food security by 6.0 percent in states with state EITC laws, with the effect being even larger (9.8 percent) in states with high EITC benefits. When examining a potential mechanism through which the two policies improve food security, we provide evidence for a joint impact on reducing poverty rates. In contrast, we find no evidence that minimum wages alone impact food insecurity or poverty rates in states without state EITC laws.

## 1 Introduction

One of the biggest societal challenges in the United States is the presence of inequalities. Some examples of these are poverty, inequalities in income, access to health and health outcomes as well as persistent food insecurity rates. Household food security is defined by all household members having access to enough food for an active, healthy life at all times (U.S. Department of Agriculture Economic Research Service 2022). According to data from the U.S. Department of Agriculture (USDA), the share of households in the U.S. that suffer from food insecurity has been above 10% since 2001 (Rabbitt et al. 2024). In 2023, 13.5% of households were food insecure, which corresponds to 18 million households and marked an increase from the previous 2 years (Rabbitt et al. 2024). Food insecurity rates differ significantly across states, with the highest food insecurity rates being in the Southern states over the last decades. During the 2021 to 2023 period, state food insecurity rates ranged from 7.4% in New Hampshire to 18.9% in Arkansas (Rabbitt et al. 2024). While it is established that socioeconomic factors such as income and employment are important determinants of food security, less is known about the role of public policy in reducing food insecurity rates.

While many studies have examined the effects of income assistance programs such as minimum wages and the Earned Income Tax Credit (EITC) on health‐related outcomes over the last decade (e.g. Averett and Wang 2013; Hoynes et al. 2015; Lenhart 2017, 2018; Wehby et al. 2019), there is much less evidence so far on whether these policies impact food insecurity. Winkler et al. (2025) provide evidence that more generous state minimum wages in the U.S. are associated with significant reductions in food insecurity among households with children, while Hasanah et al. (2024) finds similar results when examining increases in the Indonesian minimum wage. Lenhart (2023) shows that the 2009 federal expansion of the EITC improved food security levels among low‐educated households. To our knowledge, there is no evidence on whether these policies have a joint impact on food security outcomes. This study contributes to this gap in the literature. We explore the joint impact of state minimum wage and EITC laws on food security by using a period of 19 years that includes several policy changes to both policies and by analyzing a sample of individuals with at most a High School degree. Additionally, we examine the role of poverty as a potential mechanism through which minimum wages and EITC have a joint impact on food security rates.

We believe that the two policies have interaction effects in labor market responses and incomes of affected individuals. These effects on income and financial security could then reduce food insecurity rates of low‐income households. A few previous studies have made arguments for the joint effects of minimum wages and EITC. Dube and Lindner (2024) state that while the EITC is thought to encourage labor supply and thus potentially push down pre‐tax wages, minimum wages can mitigate this downward push in pay. Similarly, Rothstein and Zepperer (2020) argue that the minimum wage is an effective complement that limits any dilution and ensures that workers retain larger shares of the EITC and that the two policies are thus best used together. Having said that, the empirical evidence on the interaction between the two policies is very limited.

Neumark and Wascher (2011) examine the interaction effects between minimum wages and the EITC on labor market outcomes. The authors provide evidence for heterogenous interaction effects of the two policies. For very poor families with children, the authors find that minimum wages and EITC can be mutually enforcing and amplify the labor supply response among affected households by showing that higher minimum wages increase the positive impact of the EITC on incomes. To explain this findings, Neumark and Wascher (2011) argue that higher minimum wages increase the willingness to work to a greater extent than the EITC would alone, with this effect likely being largest for single women. When examining individuals not eligible for the EITC (e.g. teenagers, childless low‐skilled individuals), they find no evidence for positive interaction effects. Instead, they even show that minimum wages can exacerbate the potentially adverse effects of the EITC for these individuals. The authors suggest that individuals who are ineligible for the EITC can take a double hit from high minimum wages coupled with the EITC. The authors state that minimum wages reduce their employment prospects by imposing higher wage on employers, and the EITC would reduce their employment prospects through an increased labor supply of individuals eligible for the EITC.

The federal EITC, introduced in 1975, is one of the largest and most effective anti‐poverty programs in the U.S. that provides a refundable credit to low‐income households based on their income and family status. In 2023, 31 states and Washington. D.C. offer state EITC benefits in addition to the federal benefit, ranging from 4% of the federal rate in Wisconsin to 125% of the federal rate in South Carolina. In 2023, 30 states have an effective minimum wage above the federal minimum wage, which has remained unchanged at $7.25 since 2009. There is a significant variation in state minimum wage rates, with Washington having the highest rate of $17.

## 2 Data

The main objective of this study is to evaluate the joint impact of state‐level minimum wages and EITC laws on food insecurity rates. We use data from the Food Security Supplement of the Current Population Survey (CPS‐FSS), which is collected every December by the U.S. Census Bureau. Our analysis uses data from 2001 to 2019, a period during which there were 78 changes to state EITC laws and 354 changes in effective state minimum wages. Appendix Table A1 provides an overview of changes in average minimum wages and changes in EITC across the period used in our analysis. Consistent with previous research examining the effects of the EITC on well‐being (e.g. Evans and Garthwaite 2014; Hoynes et al. 2015), we restrict the sample to working age (age 18–65) individuals with at most a High School degree, a group that is more likely to be impacted by changes to income assistance programs. This provides us with a sample size of 502,195 households.

In addition to providing information on basic demographic characteristics, the survey includes data on household‐level food security. In the CPS‐FSS, food security is measured through responses to a series of 18 questions—the first 10 questions are related to adult food security status, while the last eight questions are related to child food security status. Each question is designed to measure the ability to obtain enough and nutritious food during the previous 12 months. We use a dichotomous indicator for very low household‐level food security as our main outcome variable, which has been established by the USDA and is based on the number of affirmative responses (U.S. Department of Agriculture Economic Research Service, 2022). Very low household food security is defined as having at least six or eight affirmative responses for adult‐only households and for households with children, respectively.^1^ Additionally, we use three continuous measures of food insecurity by using the sum of affirmative responses to measure food security for the entire household (out of 18), as well as for adults (out of 10) and children (out of 8) only.

We use information on state EITC laws from annual reports by the Internal Revenue Service (IRS), while we collect data on minimum wages from the Bureau of Labor Statistics (BLS). Our analysis controls for individual characteristics, including marital status, race, and gender. Additionally, we include information on time‐varying state‐level characteristics, such as unemployment rates, poverty rates, uninsurance rates as well as state TANF and SNAP benefits for a family of four.

To provide descriptive evidence for the overlap of minimum wages and EITC payments, we use data from the Annual Social and Economic Supplement (ASEC) of the CPS, which includes simulated EITC benefits for households using the Census Bureau's tax model as well as hourly wage information. Appendix Table A2 shows average EITC benefits for different groups of earners. In addition to showing statistics for low educated individuals, we also show statistics for low educated individuals who have children. The table shows that low‐educated individuals who are earning around the minimum wage are, based on the tax simulation model, substantially more likely to receive EITC benefits than those who are earning significantly more than the effective minimum wage.^2^

## 3 Methodology

Our analysis estimates difference‐in‐difference models (DID) for which states with minimum wages higher than the federal rate serve as the treatment group and those with the federal minimum wage form the control group. Using OLS models, we estimate the following specification:

(1) $$Y_{\text{ist}}=\beta_{0}+\beta_{1}\;{\text{MinWage}}_{\text{st}}+\beta_{2}\;{\text{EITC}}_{\text{st}}+\beta_{3}\;{\text{MinWage}}_{\text{st}}\;\times \;{\text{EITC}}_{\text{st}}+\beta_{4}\;X_{\text{ist}}+\beta_{5}\;Z_{\text{st}}+\lambda_{1}\;{\text{Year}}_{t}+\lambda_{2}\;{\text{State}}_{s}+\varepsilon_{\text{st}}$$

where *Y*_ist_ is an indicator that equals to one if household *i* living in state *s* reports very low food security in year *t* and zero otherwise. MinWage_st_ measures real minimum wages, while EITC_st_ is a continuous measure of state EITC benefits (state rate as a share of federal rate). The coefficient of interest is *β*_3_, which provides the interaction effect between state minimum wages and state EITC benefits. *X*_ist_ is a control for individual‐level characteristics (marital status, race, and gender). *Z*_st_ represents a set of controls accounting for potential state‐level confounding factors.^3^ Finally, both year and state fixed effects are included in the analysis.

In addition to estimating Equation (1) for all states, we examine the joint impact of minimum wages and EITC laws by separately estimating the effect of $1 increases in state minimum wages on food insecurity for (1) states with and without state EITC benefits, and (2) for states with high and low EITC rates. This can provide supportive evidence for the estimates obtained from the main specification by showing how the effects of minimum wages on food insecurity differ across states with different EITC policies. For these specifications, we estimate Equation (2), which no longer includes the interaction term between minimum wages and EITC benefits.

(2) $$Y_{\text{ist}}=\beta_{0}+\beta_{1}\;{\text{MinWage}}_{\text{st}}+\beta_{2}\;{\text{Xi}}_{\text{st}}+\beta_{3}\;Z_{\text{st}}+\lambda_{1}\;{\text{Year}}_{t}+\lambda_{2}\;{\text{State}}_{s}+\varepsilon_{\text{st}}$$

Finally, we evaluate the role of state poverty rates as a potential mechanism underlying the relationship between both policies and food security by replacing the outcome variable *Y*_ist_ with state‐level poverty rates, Poverty_st_.

## 4 Results

Table 1 shows the main results of our analysis. By estimating Equation (1), the table shows estimates for the interaction terms between state minimum wages and state EITC benefits for four different measures of food insecurity. We find statistically significant reductions in food insecurity for three of the four measures of food insecurity, which provides suggestive evidence for the joint impact of the two state policies in terms of improving food security levels of low‐educated households.

**TABLE 1:** The interaction effects of minimum wages and state EITC on food insecurity (2001–2019, at most HS degree).

	Very low food insecurity	FI score	FI score ‐ adults	FI score ‐ children
MW*EITC	−0.0012***	−0.0157***	−0.0106**	−0.0028
	(0.0004)	(0.0056)	(0.0042)	(0.0031)
MW	0.0010	−0.0134	−0.0269	−0.0008
	(0.0012)	(0.0192)	(0.0136)	(0.0111)
EITC	−0.0063	−0.6301*	−0.2183	−0.3198
	(0.0323)	(0.3686)	(0.2999)	(0.2225)
Sample mean	0.0690	1.2814	1.1517	0.4511
*N*	502,195	502,195	371,011	224,087

*Note:* The estimates show the interaction effects between state minimum wages and a continuous measure of EITC benefits. Robust standard errors, clustered by state, are shown in parentheses. All specifications control for marital status, race, gender, state poverty rates, state unemployment rates, state uninsurance rates, state TANF and SNAP benefits (for a family of four each), log state population, total state employment as well as both state and year fixed effects.

Next, we present separate estimates for the effects of $1 increases in minimum wages on food insecurity across states with different EITC laws in Table 2. Panel A estimates for states with and without state EITC laws, while Panel B provides estimates for states with high and low state EITC benefits.^4^

**TABLE 2:** The effects of minimum wages on food insecurity, by EITC Law (2001–2019, at most HS degree).

	Very low food insecurity	FI score	FI score ‐ adults	FI score ‐ children	*N*
Panel A: EITC law					
States with EITC	−0.0041***	−0.0413**	−0.0522***	−0.0166	195,143
	(0.0016)	(0.0196)	(0.0133)	(0.0114)	
Sample mean	0.0685	1.2369	1.1214	0.4316	
States without EITC	0.0004	−0.0023	−0.0257	0.0123	307,052
	(0.0018)	(0.0243)	(0.0168)	(0.0133)	
Sample mean	0.0694	1.3096	1.1734	0.4626	
Panel B: EITC size					
States with high EITC	−0.0064***	−0.0607**	−0.0563***	−0.0254	120,907
	(0.0023)	(0.0297)	(0.0194)	(0.0189)	
Sample mean	0.0656	1.2026	1.0776	0.4268	
States with low EITC	−0.0007	−0.0085	−0.0424	−0.0058	74,336
	(0.0028)	(0.0266)	(0.0315)	(0.0176)	
Sample mean	0.0731	1.2925	1.1962	0.4394	

*Note:* The estimates show the effects for a $1 increase in the effective state minimum wage. Robust standard errors, clustered by state, are shown in parentheses. With exception of the “States without EITC” sample in Panel A, all specifications control for a continuous measure of state EITC benefits (measured by state EITC rate as a share of federal rate). All specifications control for marital status, race, gender, state poverty rates, state unemployment rates, state uninsurance rates, state TANF and SNAP benefits (for a family of four each), log state population, total state employment as well as both state and year fixed effects.

We find that increases in minimum wages are only associated with statistically significant reductions in food insecurity in states that have state EITC laws in place for all four outcome variables. For these states, we find that a $1 increase in minimum wages leads to a 0.41% points decline in the likelihood of households experiencing very low food security (*p* < 0.01), which corresponds to a 5.99% reduction compared to the sample mean. We also find statistically significant decreases in the household food insecurity score as well as in the adult food insecurity score. Again, we find no impacts of minimum wages for these food insecurity measures when restricting the sample to states without EITC laws. When examining the effects on child food insecurity scores, the results follow the same overall pattern but are imprecisely estimated.

Panel B provides further evidence for the joint impact of minimum wages and state EITC laws on food security by showing that the statistically significant effect for states with EITC laws shown in Panel A is entirely driven by states with high EITC benefits (at least 10% of the federal rate). For this group of states, we find that a $1 increase in minimum wages leads to a 0.64% point decline in the likelihood that households suffer from very low food security (*p* < 0.01), which corresponds to a reduction of 9.76% compared to the sample mean. We find no statistically significant effects when restricting the sample to states low EITC benefits (less than 10% of the federal rate).^5^

Next, we calculate the predicted level of food insecurity for different combinations of minimum wages and state EITC laws. Following Winkler et al. (2025), we estimate separate estimated means for the federal and the maximum state minimum wage and using the same three categories of EITC generosity (none, small and high) as in Table 2. The predicted levels of very low food security are presented in Appendix Table A3. While EITC generosity does not appear to influence food insecurity rates in states that have the federal minimum wage, we find that higher EITC rates can improve food security in states that have the maximum minimum wage. The estimate rate of very low food security is 8.71% in states with a maximum minimum wage but without any state EITC law, whereas it is 5.94% in states with both the maximum minimum wage and a high EITC rate. These estimates provide further suggestive evidence for the joint impact of the two policies in reducing food insecurity rates.

While we allow states with different EITC laws to change groups after implementing policy changes in the analysis shown in Table 2, we re‐estimate these specifications after creating a balanced panel that does not change EITC laws. Appendix Table A4 presents the estimates for this analysis. We find that a $1 increase in minimum wages decreases the likelihood of households experiencing very low food security by 0.45% points (*p* < 0.05) in states that always have state EITC laws during our sample period. In contrast, we find statistically significant (*p* < 0.05) positive effect in states that never had state EITC laws. This further supports our main findings of a joint impact of the two policies. Interestingly, we also find that the effect of minimum wages on food insecurity is larger in states that always had a low state EITC rate in comparison to those that always had a high state EITC rate.

In an additional robustness check, we repeat our main analysis restricting the sample to households with at least some college education, a group that should be less impacted by changes to minimum wages and EITC rates. Consistent with this, Appendix Table A5 shows no joint impact of the two policies on the likelihood of experiencing very low food security when using the interaction term in the analysis. When estimating the effect of $1 increases in minimum wages across states with different EITC laws, we also find that there is no impact on food security for the high‐educated sample, independent of whether states have a (high) EITC rate or not.

In Table 3, we present separate estimates by household types (households with and without children as well as single and married households) and race (whites and blacks).^6^ Overall, the results suggests that the joint impact of minimum wages and state EITC laws exists among all the six groups that we examine, although we observe differences in the magnitude of the impacts. We find that our observed reduction in food insecurity is mainly driven by single households, which is in line with both evidence from the EITC literature showing that single households benefit more from increases to the credit (e.g. Eissa and Liebman 1996; Meyer and Rosenbaum 2000, 2001) and evidence on the interaction effects of the two policies (Neumark and Wascher 2011). Table 3 also shows that the effects are substantially larger among black than white households, which could be explained by a higher proportion of Blacks being eligible for EITC benefits (e.g. Batra et al. 2021) and by higher food insecurity rates are among black households (sample mean of 10.46% for Blacks, 6.18% for Whites).

**TABLE 3:** The effects of minimum wages and EITC laws on food insecurity, by HH type and race.

	Very low food security					
	HH type				Race	
	HH with children	HH without children	Single	Married	White	Black
States with EITC	−0.0033*	−0.0046**	−0.0060**	−0.0029*	−0.0031*	−0.0128**
	(0.0020)	(0.0019)	(0.0028)	(0.0016)	(0.0016)	(0.0055)
Sample mean	0.0550	0.0787	0.0985	0.0426	0.0618	0.1046
States without EITC	−0.0002	0.0007	0.0009	−0.0008	0.0010	−0.0027
	(0.0021)	(0.0023)	(0.0032)	(0.0015)	(0.0019)	(0.0074)
Sample mean	0.0561	0.0802	0.1002	0.0438	0.0630	0.1072

*Note:* The estimates show effects for a $1 increased in the effective state minimum wage. Robust standard errors, clustered by state, are shown in parentheses. All specifications control for marital status, race, gender, state poverty rates, state unemployment rates, state uninsurance rates, state TANF and SNAP benefits (for a family of four each), log state population, total state employment as well as both state and year fixed effects.

Next, we examine the role of poverty as a potential mechanism through which the two income assistance programs reduce food insecurity. Table 4 shows that there is a joint impact on poverty rates, which is consistent with the findings for food insecurity. We find a statistically significant reduction in poverty rates (*p* < 0.10) when including the interaction terms between state minimum wages and EITC benefits. Like our main analysis, the joint impact of the two policies is confirmed when separately estimating the effect of minimum wage increases across EITC laws. In states that have EITC laws in place, we find that a $1 increase in the effective minimum wage leads to a 0.30% point reduction in state poverty rates (*p* < 0.05), whereas this effect increases to 0.34% point when restricting the sample to states with high EITC rates (*p* < 0.05). These effects correspond to reductions in poverty rates of 2.46 and 2.85% compared to the sample means, respectively. Overall, the estimates in Table 4 suggest that the positive impact of generous state minimum wages and EITC laws on food security among low‐educated households is partly due to the joint impact of these programs on poverty rates. This finding is consistent with evidence by Rothstein and Zepperer (2020) who show that coupling EITC expansions with high minimum wages reduces poverty.

**TABLE 4:** The effects of minimum wages and EITC laws on poverty rates.

	State poverty rate	*N*
Panel A: Interaction effect		
MW*EITC	−0.5951*	969
	(0.3115)	
MW	0.0277	
	(0.1384)	
EITC	6.4481**	
	(3.0966)	
Sample mean	12.88	
Panel B: EITC law		
States with EITC	−0.3018**	407
	(0.1191)	
Sample mean	12.29	
States without EITC	0.1266	562
	(0.1963)	
Sample mean	13.32	
Panel C: EITC size		
States with high EITC	−0.3412**	269
	(0.1451)	
Sample mean	11.96	
States with low EITC	−0.1095	138
	(0.2602)	
Sample mean	12.94	

*Note:* The estimates show the effects for a $1 increase in the effective state minimum wage. Robust standard errors, clustered by state, are shown in parentheses. With exception of the “States without EITC” sample in Panel B, all specifications control for a continuous measure of state EITC benefits (measured by state EITC rate as a share of federal rate). All specifications control for state unemployment rates, state uninsurance rates, state TANF and SNAP benefits (for a family of four each), log state population, total state employment as well as both state and year fixed effects.

## 5 Conclusion

Our study provides evidence that minimum wages and state EITC laws have a positive joint impact on food security. The findings add to a small number of papers demonstrating that pairing state EITC laws with generous minimum wages can lead to improvements in well‐being. Hurst et al. (2022) show that the two policies combined positively impact employment, labor income and welfare, while Lenhart and Chakraborty (2024) find that that the programs have a positive joint impact on measures of population health, such as reducing suicide and assault rates. Given the persistent issue of high food insecurity rates in the U.S., especially among households with at most a High School degree, our study presents evidence that combining generous minimum wages with high state EITC rates can help improve food security levels of these households. Furthermore, we show that reductions in poverty rates are a likely channel through which the two income assistance programs impact food security. The findings are consistent with claims that enacting or improving both policies in tandem will reach more workers than either one on its own (Williams et al. 2020). Our results suggest that, to improve the well‐being of the population and reduce existing societal inequalities, the federal minimum wage should be increased for the first time since 2009, and that more states should consider introducing state EITC laws.

## Conflicts of Interest

The authors declare no conflicts of interest.

## Data Availability

The data that support the findings of this study are available from the corresponding author upon reasonable request.

## Endnotes

^1^
This threshold has also been used in previous studies examining food security with the same set of 18 questions.

^2^
Additionally, when comparing the mean hourly wages for low‐educated individuals who receive any EITC ($11.28) and those who do not ($14.10), we find a large difference, which furthermore provides suggestive overlap than there is an overlap between the two policies. The difference in mean wages is even larger for low‐educated individuals with children ($11.39 and $15.00). Furthermore, Rothstein and Zepperer (2020) argue that nowadays many more EITC workers are paid the minimum wage than in the 1990s because of many states and localities having raised the real value of their local minimum wages in recent decades.

^3^
The state‐level controls include state unemployment rates, the share of state population below the age of 65 without any insurance coverage, state poverty rates as well as state TANF and SNAP benefits for a family of 4, log state population and total state employment. It should be noted that despite controlling for other state policies and characteristics, we might not be able to fully isolate the role of minimum wage and EITC policies since these might also be correlated with other safety net policies.

^4^
In deciding for the appropriate cutoff point between high and low state EITC benefits, we follow the approach in the literature and use 10 percent of the federal rate, which is the median value of state EITC benefits over our sample period (Markowitz et al. 2017; Lenhart 2019).

^5^
Dube et al. (2010) show that traditional two‐way fixed effects can lead to a downward bias when estimating the effects of minimum wages on earnings and employment. The authors suggest that using a congruous county, cross‐state border design is a preferred method when analysing the impact of minimum wages. Having said that, Dube et al. (2010) also mention that the CPS is not well suited for the cross‐state border design due to small sample sizes and the lack of local identifiers. Given that food security information is only available in December, the small sample issue is even more persistent and does not allow us to implement the cross‐border design. It should however be noted that our estimates might suffer from downward bias following Dube et al. (2010).

^6^
The sample sizes for other racial groups (e.g. Hispanics) were too small to allow examining them.

## Appendix A

**TABLE A1:** Minimum wages and state EITC rates over time.

Year	Minimum wage (nominal)	# Of state EITC changes	State EITC (% of federal rate)	
			All states	States with EITC
2001	5.41 (0.49)	5	0.05 (0.09)	0.16 (0.10)
2002	5.48 (0.61)	5	0.05 (0.09)	0.17 (0.10)
2003	5.51 (0.65)	6	0.05 (0.10)	0.16 (0.11)
2004	5.55 (0.70)	0	0.05 (0.10)	0.16 (0.11)
2005	5.72 (0.75)	1	0.06 (0.10)	0.17 (0.11)
2006	5.92 (0.84)	3	0.06 (0.11)	0.17 (0.11)
2007	6.58 (0.68)	4	0.06 (0.11)	0.17 (0.11)
2008	7.00 (0.52)	8	0.07 (0.11)	0.17 (0.11)
2009	7.43 (0.34)	4	0.07 (0.11)	0.17 (0.11)
2010	7.46 (0.36)	2	0.07 (0.11)	0.17 (0.11)
2011	7.50 (0.40)	2	0.08 (0.11)	0.18 (0.11)
2012	7.55 (0.44)	3	0.08 (0.11)	0.17 (0.11)
2013	7.58 (0.46)	2	0.08 (0.11)	0.17 (0.11)
2014	7.80 (0.66)	6	0.08 (0.11)	0.16 (0.10)
2015	8.00 (0.78)	2	0.08 (0.11)	0.15 (0.11)
2016	8.25 (1.08)	8	0.15 (0.24)	0.29 (0.28)
2017	8.44 (1.24)	3	0.15 (0.25)	0.30 (0.28)
2018	8.71 (1.57)	7	0.16 (0.25)	0.30 (0.27)
2019	9.23 (2.04)	7	0.17 (0.25)	0.32 (0.26)
All years	7.05 (1.44)	78	0.08 (0.15)	0.20 (0.17)

*Note:* Standard deviations are shown in parentheses. Data for minimum wages is collected from the Bureau of Labor Statistics (BLS), whereas information on state EITC laws is obtained from annual reports by the Internal Revenue Service (IRS).

**TABLE A2:** Descriptive statistics ‐ minimum wage and EITC overlap.

	Low education		Low education, with children	
	Total EITC	Any EITC	Total EITC	Any EITC
At or below MW	335.99	0.1702	876.22	0.3189
At or below 1.10*MW	349.43	0.1754	937.27	0.3413
At or below 1.20*MW	365.24	0.1821	976.83	0.3589
At or below 1.30*MW	376.88	0.1855	997.85	0.3654
Above 2*MW	209.38	0.1020	514.89	0.2071
Above 3*MW	73.39	0.0456	124.71	0.0654
Above 4*MW	64.36	0.0367	103.81	0.0488

**TABLE A3:** Estimated likelihood for very low food insecurity.

	Estimated means
Federal MW, no EITC	0.0740
Federal MW, low EITC	0.0718
Federal MW, high EITC	0.0727
Maximum MW, no EITC	0.0871
Maximum MW, low EITC	0.0953
Maximum MW, high EITC	0.0594

*Note:* Estimates are obtained from models using robust standard errors, clustered by states as well as the margins command on Stata SE version 18.0. Low state EITC rates are less than 10% of the federal rate, whereas high EITC rates are at least 10% of the federal rate. The models control for marital status, race, gender, state poverty rates, state unemployment rates, state uninsurance rates, state TANF and SNAP benefits (for a family of four each), log state population, total state employment as well as both state and year fixed effects.

**TABLE A4:** The effects of minimum wages on food insecurity, by constant EITC laws (2001–2019, at most HS degree).

	Very low food insecurity	FI score	FI score ‐ adults	FI score ‐ children	*N*
Panel A: EITC law					
Always EITC states	−0.0045**	−0.0249	−0.0407*	−0.0074	126,782
	(0.0021)	(0.0305)	(0.0206)	(0.0169)	
Sample mean	0.0399	0.7500	0.6843	0.2645	
Never EITC states	0.0048**	0.0380	−0.0045	0.0238	204,579
	(0.0023)	(0.0309)	(0.0232)	(0.0172)	
Sample mean	0.0479	0.9117	0.8174	0.3127	
Panel B: EITC size					
Always high EITC states	−0.0034	−0.0150	−0.0140	0.0042	79,345
	(0.0026)	(0.0343)	(0.0208)	(0.0232)	
Sample mean	0.0380	0.7199	0.6509	0.2602	
Always low EITC states	−0.0076*	−0.1143**	−0.0843	−0.0180	24,812
	(0.0064)	(0.0505)	(0.0568)	(0.0155)	
Sample mean	0.0491	0.8709	0.8136	0.2982	

*Note:* The estimates show the effects for a $1 increase in the effective state minimum wage. Robust standard errors, clustered by state, are shown in parentheses. With exception of the “Never EITC states” sample in Panel A, all specifications control for a continuous measure of state EITC benefits (measured by state EITC rate as a share of federal rate). All specifications control for marital status, race, gender, state poverty rates, state unemployment rates, state uninsurance rates, state TANF and SNAP benefits (for a family of 4 each), log state population, total state employment as well as both state and year fixed effects.

**TABLE A5:** The effects of minimum wages and EITC on food insecurity (2001–2019, at least some college).

	Very low food insecurity	*N*
Panel A: Interaction term		
MW*EITC	−0.0001	753,624
	(0.0003)	
Sample mean	0.0291	
Panel B: EITC law		
States with EITC	−0.0002	328,124
	(0.0006)	
Sample mean	0.0275	
States without EITC	−0.0004	425,500
	(0.0006)	
Sample mean	0.0302	
Panel C: EITC size		
States with high EITC	0.0001	219,313
	(0.0007)	
Sample mean	0.0254	
States with low EITC	−0.0025	108,811
	(0.0016)	
Sample mean	0.0319	

*Note:* The estimates show the effects for a $1 increase in the effective state minimum wage. Robust standard errors, clustered by state, are shown in parentheses. With exception of the “States without EITC” sample in Panel B, all specifications control for a continuous measure of state EITC benefits (measured by state EITC rate as a share of federal rate). All specifications control for marital status, race, gender, state poverty rates, state unemployment rates, state uninsurance rates, state TANF and SNAP benefits (for a family of four each), log state population, total state employment as well as both state and year fixed effects.
